# DAILY AND TECHNOLOGICAL CHALLENGES AND NEEDS IN OLDER AGES: A MIXED METHODS STUDY

**DOI:** 10.1093/geroni/igae098.0561

**Published:** 2024-12-31

**Authors:** Michelle Cruz, Li Chu, Laura Paola Gomezjurado Gonzalez, Ruohan Zhang, Jiajun Wu, Li Fei-Fei, Laura Carstensen

**Affiliations:** Stanford University, Stanford, California, United States; Stanford University, Stanford, California, United States; Stanford University, Stanford, California, United States; Stanford University, Stanford, California, United States; Stanford University, Stanford, California, United States; Stanford University, Stanford, California, United States; Stanford University, Stanford University, California, United States

## Abstract

Modern societies face two trends that are seemingly unrelated yet concurrently impacting our lives: population aging and technological advancements. Both trends are happening rapidly in today’s society, and in combination raise an important question: How can we guide future technologies and their deliveries to better suit older adults’ needs so older adults are not “left behind” in the fast-changing cultural shifts due to technological advancements? A growing body of literature captures technological needs and problems among older adults. Yet, many of these studies used relatively small sample sizes consisting of relatively young older adults who are homogeneous in terms of race and ethnicity to represent all older adults. The present study utilizes a mixed methods design to ensure qualitative findings are generalizable. We recruited a racially diverse sample of Asian, Black/African, Hispanic, and White community-dwelling older adults over the age of 75 from the San Francisco Bay Area to form eight focus groups. Participants freely discussed their daily challenges and needs, and how technology may meet these needs. With manual coding and natural language processing techniques, we identified close to 30 areas where technologies may contribute to daily life. Findings from the qualitative sessions and a follow-up survey will be presented. Our findings have the potential to inform service providers, government, technologists, and entrepreneurs how they can better support older adults who are independent living but have started to observe difficulties.

